# Molecular Fingerprint Imaging to Identify Dental Caries Using Raman Spectroscopy

**DOI:** 10.3390/ma13214900

**Published:** 2020-10-31

**Authors:** Nao Miyamoto, Tetsuya Adachi, Francesco Boschetto, Matteo Zanocco, Toshiro Yamamoto, Elia Marin, Shota Somekawa, Ryutaro Ashida, Wenliang Zhu, Narisato Kanamura, Ichiro Nishimura, Giuseppe Pezzotti

**Affiliations:** 1 Department of Dental Medicine, Graduate School of Medical Science, Kyoto Prefectural University of Medicine, Kamigyo-ku, Kyoto 602-8566, Japan; n-miya@koto.kpu-m.ac.jp (N.M.); boschetto.cesc@gmail.com (F.B.); yamamoto@koto.kpu-m.ac.jp (T.Y.); elia-marin@kit.ac.jp (E.M.); kanamura@koto.kpu-m.ac.jp (N.K.); 2 Infectious Diseases, Kyoto Prefectural University of Medicine, Kamigyo-ku, 465 Kajii-cho, Kyoto 602-8566, Japan; 3 Dentistry, Kyoto Prefectural Rehabilitation Hospital for Mentally and Physically Disabled, Naka Ashihara, Johyo City, Kyoto 610-0113, Japan; 4 Ceramic Physics Laboratory, Kyoto Institute of Technology, Sakyo-ku, Matsugasaki, Kyoto 606-8585, Japan; matteo.zanocco@gmail.com (M.Z.); wlzhu@kit.ac.jp (W.Z.); 5 Department of Immunology, Graduate School of Medical Science, Kyoto Prefectural University of Medicine, Kamigyo-ku, 465 Kajii-cho, Kyoto 602-8566, Japan; 6 ShinSei Co., Ltd., Hiramori Okubo-cho, Uji-shi, Kyoto 611-0033, Japan; s.somekawa@mold-shinsei.co.jp (S.S.); r.ashida@mold-shinsei.co.jp (R.A.); 7 Division of Oral Biology and Medicine, The Jane and Jerry Weintraub Center for Re-constructive Biotechnology, UCLA School of Dentistry, Los Angeles, CA 90095, USA; inishimura@dentistry.ucla.edu; 8 Division of Advanced Prosthodontics, The Jane and Jerry Weintraub Center for Re-constructive Biotechnology, UCLA School of Dentistry, Los Angeles, CA 90095, USA

**Keywords:** dentistry, Raman spectroscopy, caries, laser fluorescence

## Abstract

Tooth loss impairs mastication, deglutition and esthetics and affects systemic health through nutritional deficiency, weight loss, muscle weakness, delayed wound healing, and bone fragility. Approximately 90% of tooth loss is due to dental caries and periodontal disease. Accordingly, early treatment of dental caries is essential to maintaining quality of life. To date, the clinical diagnosis of dental caries has been based on each dentist’s subjective assessment, but this visual method lacks objectivity. To improve diagnostic ability, highly sensitive quantitative methods have been developed for the diagnosis and prevention of dental caries and are gradually becoming a mandatory item in modern dentistry. High-resolution Raman spectroscopy is a suitable tool for recognizing the subtle structural changes that occur in dental enamel in already developed or, more importantly, incipient dental caries. Raman analysis could soon emerge as a breakthrough in dentistry because of its high diagnostic sensitivity. In this study, we build upon our previous findings in a new analysis of dental caries using Raman spectroscopy imaging and discuss the possibility of using Raman photonic imaging in support of objective diagnostics in dentistry. Our findings support the Raman method of caries detection in comparison with other conventional or new approaches.

## 1. Introduction

Raman spectroscopy is an analytical method that consists of irradiating a sample with a visible laser source, then determining the type and condition of the substance based of the characteristics of the Raman scattered light that is generated. Evaluations of crystallinity, residual stress analyses, and chemical assessments of molecular structures can thus be performed without contact, damage, or radiation exposure. Since Raman spectroscopy allows analysis at the molecular level without requiring fluorescent probes, fixation, or other types of manipulation, it has recently received attention for its potential applications in several fields of medical science, including dentistry [1]. Various methods of dental analysis using Raman spectroscopy have recently been reported, such as the detection of trace elements in the enamel [2] and the detection of dentin formation in the dental pulp tissue [3]. However, Raman spectroscopy as a means of diagnosing dental caries has long been considered difficult. The reason for this is that the sites of dental caries generate fluorescence noise that exceeds the Raman light and varies according to measurement site and configuration. Recently, however, the combination of Raman spectroscopy with optical coherence tomography and fluorescence subtraction methods using multichannel lock-in detection have also been reported in order to avoid fluorescence interference [4,5]. Furthermore, modern devices have enabled us to collect Raman data at various measurement sites and configurations and to construct highly reliable algorithms with suitable precision [6]. When such algorithms are used as diagnostic tools, as we have recently reported, Raman spectroscopy can be used as a novel method for the early diagnosis of dental caries [1]. Moreover, Raman spectroscopy allows for the detection of a threshold state of irreversible structural change at the very early stages of tooth enamel demineralization, as caused, for example, by carbonated beverages (e.g., Coca-Cola^TM^) [7]. Based on the technique’s confirmed analytical sensitivity, we firmly believe that, in the near future, Raman spectroscopy will allow for a prompt and efficient evaluation of enamel locations that are likely to develop dental caries with high probability.

Dental caries develop as a result of the demineralization of hydroxyapatite, which is in turn a consequence of the acidic environment produced by *Streptococcus mutans*, the causative organism of dental caries [8,9,10]. X-ray radiographic imaging, currently the most popular tool in clinical settings for dental diagnosis, evaluates the current condition (simultaneity), whereas Raman spectrometry allows for evaluation of the condition before the onset of disease (predictivity). This unique predictive capacity, which could lead to a paradigm shift in dental diagnostic methods, fits in well with the recently popular concept of preemptive medicine. Preemptive medicine is a new medical paradigm in which a medical treatment is adjusted to suit the mechanism of occurrence and progression of the pathological condition and its etiology [11]. This goal is achieved by predicting in advance, and with high accuracy, that a disease will develop (predictive diagnosis), or by determining the precise diagnosis before the onset of disease (precise medicine) to prevent or at least delay disease occurrence. To successfully incorporate preemptive medicine into the dental field, highly sensitive diagnostic equipment allowing for the early detection of dental caries will be required. Using Raman spectrophotometry to perform predictive diagnosis with high accuracy (and reliability) at the initial stages prior to the onset of dental caries, and thus intervening before disease development to prevent dental caries, is now technologically possible. In addition, Raman spectrophotometry does not require exposure to radiation. Gonzalez et al. have previously reported that, in Japanese subjects, 3.2% of cancers were due to radiation used in medical diagnostic tests [12]. In addition, Pearce et al. [13] have reported that, among children who have been exposed to radiation used in medical diagnostic tests, the risk of subsequently developing leukemia and brain tumors could reach as much as three times higher than normal. Currently, diagnostic imaging in dentistry consists mostly of bitewing and panoramic radiography as well as CT imaging [14]. Because Raman spectroscopy eliminates the need for radiation exposure, it is believed to be a promising diagnostic tool. Moreover, diagnosis of non-cavitated proximal caries has long been a challenging task when conventional radiographic methods are used [15]. Accordingly, efforts have been made to develop more sensitive diagnostic tools for detecting initial lesions in populations with high caries risk and prevalence.

Among these innovative approaches are the proposed transillumination-based caries detection methods, which employ either a narrow-beam white light [16], as in fiber-optic transillumination (FOTI) and digital imaging fiber-optic transillumination (DIFOTI), or a near-infrared source [17], as in near-infrared transillumination (NIRT). Both of these methods are based on the same principle, which exploits the differences in photon scattering/absorption between healthy and disrupted enamel crystals, imaging the latter in dark shadow. Caries detection by laser fluorescence (LF) [18] exploits the semi-empirical finding that a monochromatic red light applied to a tooth location with caries can excite a strong (intrinsic) fluorescence emission because of changes in the tooth tissue. This increase in fluorescence is mainly due to a bacterial photosensitive pigment present in caries tissue, but it could also be contributed by other factors related to oral hygiene. The FOTI/DIFOTI, NIRT, and LF methods have all been commercialized in dentistry, and their caries detection sensitivity/specificity is currently under examination [19]. Nowadays, the LF method is perhaps the most advanced radiation-free method available to enhance early identification of caries and to follow their development through regular successive examinations.

The present study proposes to utilize Raman spectroscopy as an alternative radiation-free method that is superior in sensitivity to LF. We anticipate that the Raman method could play a key diagnostic role in future dentistry now that Raman devices have a sufficiently high sensitivity to be suitable for both diagnostic and preventive purposes.

We have previously established working algorithms for Raman spectrometry in the diagnosis of surface caries in the enamel structure [6], but its usefulness for caries in the dentin structure has not yet been examined. Dentin, a hard tissue constituting a major component of the tooth, is located between the pulp cavity and the enamel or cementum. The chemical composition of dentin differs greatly from that of enamel, being composed of 70% hydroxyapatite (which makes up 96% of enamel) and 20% collagen fibers. Dentin is softer than enamel; this is believed to be the main reason why dental caries progress rapidly after the dentin is reached. During caries development, when the dentin caries reaches the pulp, severe spontaneous pain may occur as a result of complication by pulpitis. In addition, at that stage of disease development, the dental crown is likely to collapse as a result of the fragility of the tooth, which may ultimately lead to tooth extraction. To avoid this outcome, dental caries must be detected and treated in their very earliest stage, namely, the stage of early enamel demineralization.

In this study, we apply Raman spectroscopy to visualizing and quantifying the extent of dental caries buried in the subsurface of the dentin structure. This study is in line with our previous studies of caries in the enamel structure, but this is our first study treating caries that are hardly diagnosable by either visual examination or conventional methods such as X-rays. Applying the method described in this study may help prevent tooth loss and preserve patients’ quality of life. Raman photonic diagnostics is the key to the successful application of preemptive medicine in dentistry.

## 2. Materials and Methods

### 2.1. Teeth

Following consent from the donors, extracted human teeth were collected from the Department of Dentistry at University Hospital, Kyoto Prefectural University of Medicine or the Department of Dentistry, Kyoto Prefectural Rehabilitation Hospital For Mentally and Physically Disabled under the Review Boards (ERB-C-136)(RBMR17) (RBMR19).

### 2.2. Digital Microscopy Experiments

Each sample was examined by using a digital microscope (VHX-2000, Keyence, Osaka, Japan) in order to screen and evaluate the surface morphology of caries and cracks. Digital photographs were taken on fully focused images at ×100 magnification using depth composition function. The images were treated with software provided by the maker of the instrument.

### 2.3. Radiographic Examination

X-ray images of individual samples were obtained with an intraoral X-ray unit (Digora Optime UV, KaVo Dental Systems Co., Ltd., Berlin, Germany). The studied teeth were also subjected to micro CT imaging (inspeXio SMX-225CT, Shimadzu Co., Ltd., Kyoto-shi, Japan and/or CT Lab HX, Rigaku Co., Ltd., Tokyo, Japan). The reconstructed data sets were examined with three-dimensional data analysis software (TRI/3-D-BON, Ratoc System Engineering Co., Ltd., Tokyo, Japan and/or Materialise Mimics, Materialise NV, Leuven, Belgium).

### 2.4. Fluorescence Assessments of Caries

The caries diagnostic device DIAGNOdent™ pen (KaVo Dental, GmbH, Biberach, Germany) was employed for comparison at the same locations where Raman spectra were collected. This is a commercially available device that operates by irradiating the locations of suspected caries with laser light at 655 nm to produce fluorescent excitation at wavelengths of 670–800 nm [20,21,22,23,24,25]. The fluorescence method required preliminary calibrations, which were performed in accordance with the manufacturer’s instructions.

According to these instructions, DIAGNOdent values between 0 and 5 indicated healthy dentin, those between 5 and 40 were representative of initial caries requiring follow-up, and those >40 caries indicated requiring invasive treatment. In our analyses of enamel with suspected caries, we placed the narrow tip of the DIAGNOdent pen perpendicular to the tooth surface and shifted it parallel to the enamel surface to obtain line scans.

### 2.5. Raman Spectroscopy Experiments

Raman spectroscopic experiments were performed with a confocal Laser Raman microscope (RAMANtouch, Nanophoton Co., Ltd., Osaka, Japan). The excitation source was a green laser beam (532 nm) operating with a power of 200 mW. The power was set to an appropriate value by preliminary adjustment of ND filter and exposure time of the measurement. The microprobe used a 10x objective lens with a numerical aperture of 0.5. Confocal experiments were carried out with a pinhole aperture of 100 µm. The Raman instrument averaged data acquired at each spectral location over 4 successive measurement. Spectral deconvolution has been performed by means of an automatic fitting algorithm enclosed in the commercially available computational package (OriginLab 9.0; OriginLab Corporation, Northampton, MA, USA), using mixed Gaussian/Lorentzian curves. For the baseline, a polynomial function as been use with the same parameter for all the average spectra. Regarding the Raman images, 400 spectra were concurrently acquired on a straight line and images were generated by translating the line along a perpendicular direction.

## 3. Results

### 3.1. Digital Microscopy Experiments and Radiographic Examinations

**Figure 1:**
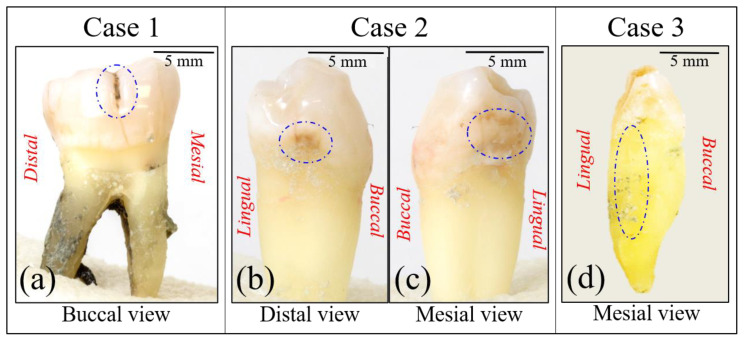
Photographs taken with a standard digital camera of the three selected cases of explanted teeth examined in this paper, referred to as Cases 1, 2, and 3 (in (**a**), (**b**,**c**), and (**d**), respectively). Dotted circles indicate cavities or fracture.

Figure 1a–d show photographs taken with a standard digital camera of the three selected cases of extracted teeth examined in this paper, referred to as Cases 1, 2, and 3, respectively. In Case 1, we observed a linear discoloration of the enamel surface on the palatal side of the crown, while in Case 2 cavities could be observed on both mesial and distal views of the crown. Case 3 represented a different pattern of vertical fracture of the dentin in the mesial view. These cases were selected because they share the common feature of visible cracking without obvious cavitation, but also because their lesions were particularly difficult to interpret: lesions like these can sometimes be hiding caries of relatively large volume that are buried under the cracked subsurface and thus hidden from direct view in visual inspection.

**Figure 2:**
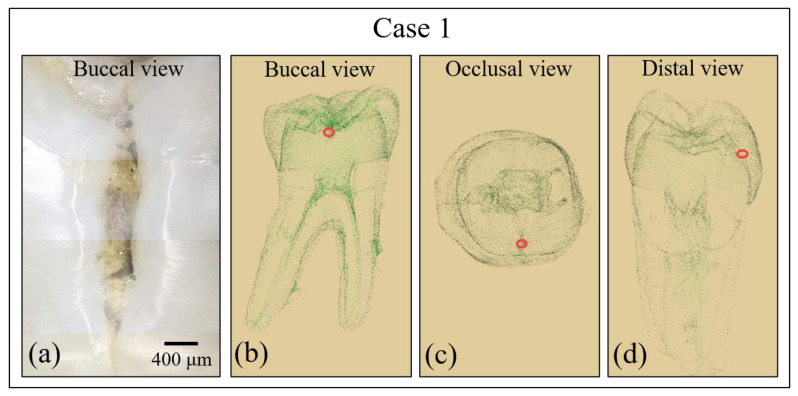
(**a**) Digital microscopic image of the crack damage in the tooth sample in Case 1. Image is at 100× magnification. (**b**–**d**) The location of the cavity and fracture in a 3D reconstructed micro-CT image in buccal (**b**), occlusal (**c**), and distal (**d**) views.

**Figure 3:**
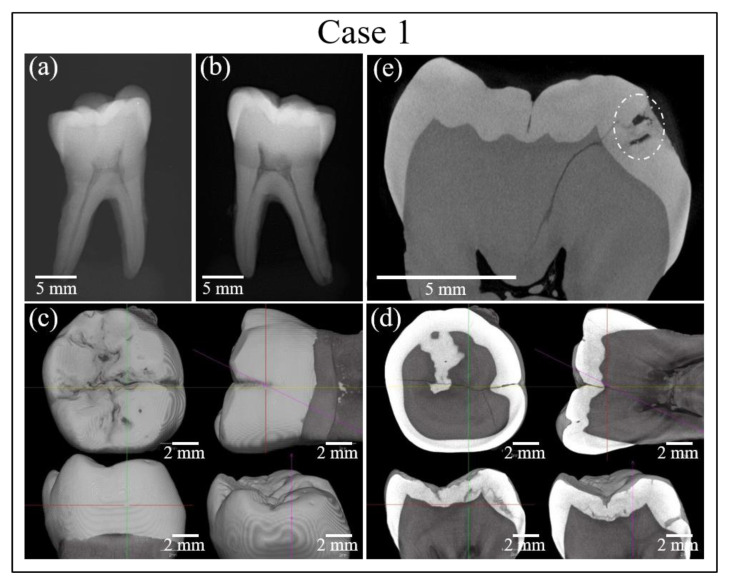
(**a**,**b**) Results of conventional X-ray analyses of the tooth sample in Case 1. (**c**–**e**) High-resolution micro-CT scanning examinations of the same sample. Dotted circle indicates cavity.

Figure 2 and Figure 3 show the results of additional analyses of the sample in Case 1. Figure 2a shows a digital microscopic image of the discolored area in the tooth sample, while Figure 2b–d show the location of the cavity as assessed by micro-CT and reconstructed into a 3D image. Figure 3a,b show the results of conventional X-ray analyses, while Figure 3c–e show high-resolution micro-CT scanning examinations. However, analyses by conventional methods gave no clear indication regarding the possible presence of buried caries, which remained hardly detectable by digital microscopy (cf. red circles in inset to Figure 2b–d) and completely invisible in conventional X-ray radiographs (cf. Figure 3a,b). Conversely, high-resolution CT-scanning clearly detected the presence of sub-surface caries (cf. encircled region in Figure 3e). It should be noted, however, that, due to radiation-related safety issues, in-vivo micro-CT imaging exams are usually performed only when there is a clear medical benefit. This method is therefore not a common choice for routine caries detection.

**Figure 4:**
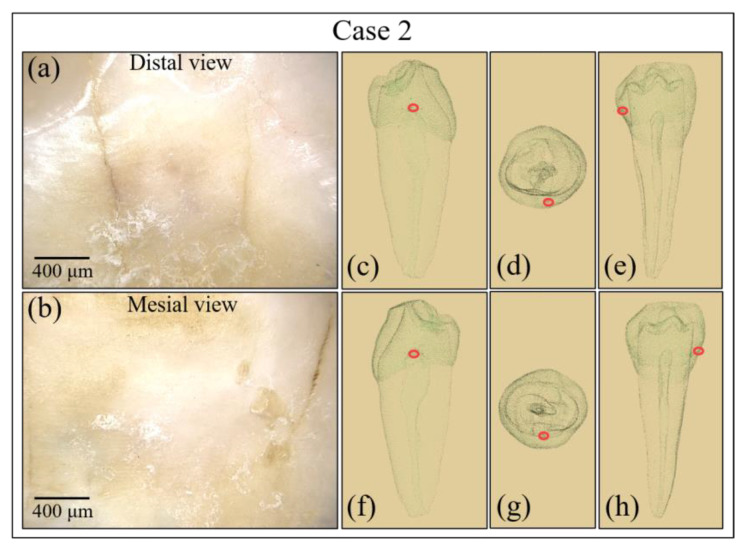
Optical views of the crack damage in the tooth sample of Case 2 on the distal (**a**) and mesial (**b**) sides; observation of cavities and fracture with a digital microscope from the distal. Images are at 100× magnification. (**c**–**e**) and mesial (**f**–**h**) sides.

**Figure 5:**
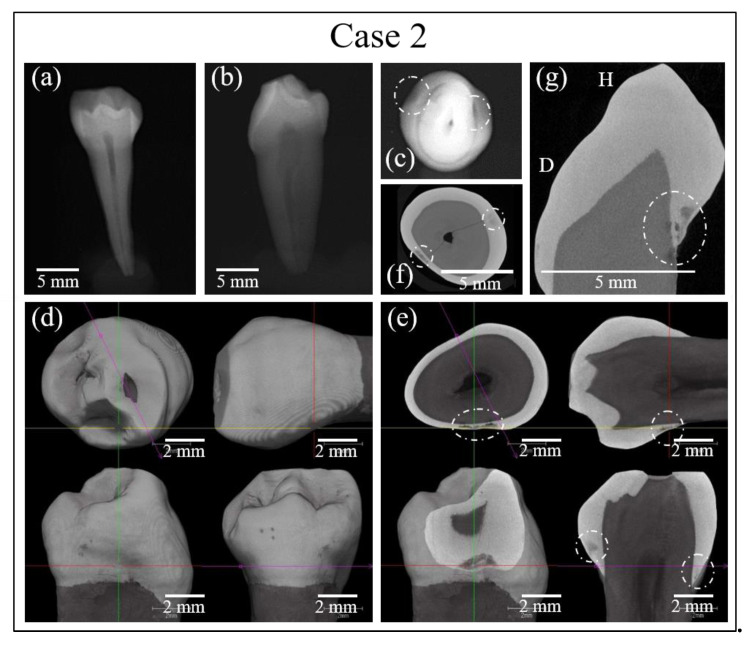
(**a**–**c**) Results of conventional X-ray analyses in the tooth sample of Case 2. (**d**–**g**) High-resolution micro-CT scanning examinations of the same sample. Dotted circles indicate cavities.

Similar analyses were performed on the Case 2 tooth sample and a diagnostic conclusion was reached. The results are shown in Figure 4 (enlarged optical micrographs in (Figure 4a,b), and 3D reconstructed micro-CT images in (Figure 4c–h)) and 5 (conventional X-ray images in (Figure 5a–c), and high-resolution micro-CT scanning in (Figure 5d–g)). As seen, buried caries were hardly detectable by optical imaging, while conventional X-rays detected cavities only in images taken from directly above the tooth crown (cf. encircled regions in Figure 5c). Note, however, that this direction of X-ray shooting is hardly applicable in vivo. Buried cavities were also clearly detected by micro-CT scanning (cf. encircled regions in Figure 5e–g), which, as mentioned above with regard to Case 1, is not a common diagnostic technique.

**Figure 6:**
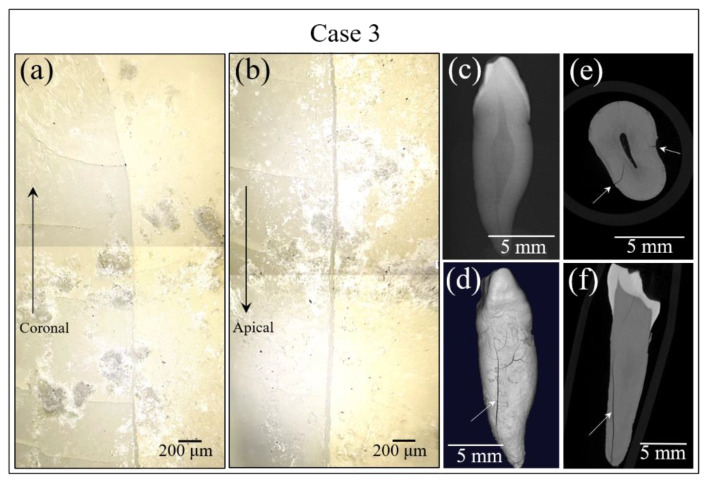
Digital microscope images of cracking locations in (**a**) and (**b**), Images are at 100× magnification. conventional X-ray image in (**c**), and overall micro-CT scan images in (**d**–**f**) for the tooth sample in Case 3. Arrows indicate fractures.

Figure 6 summarizes the set of analyses performed on the Case 3 sample. This sample was different from the previous two analyzed because cracks had propagated into the dentin region. Figure 6a,b show optical images of cracking locations. These images gave no information about the possible presence of buried caries. In this case, no sub-surface cavities could be detected either by conventional X-rays (not shown) or by micro-CT scanning (cf. in Figure 6c,d).

### 3.2. Laser Fluorescence Analysis

**Figure 7:**
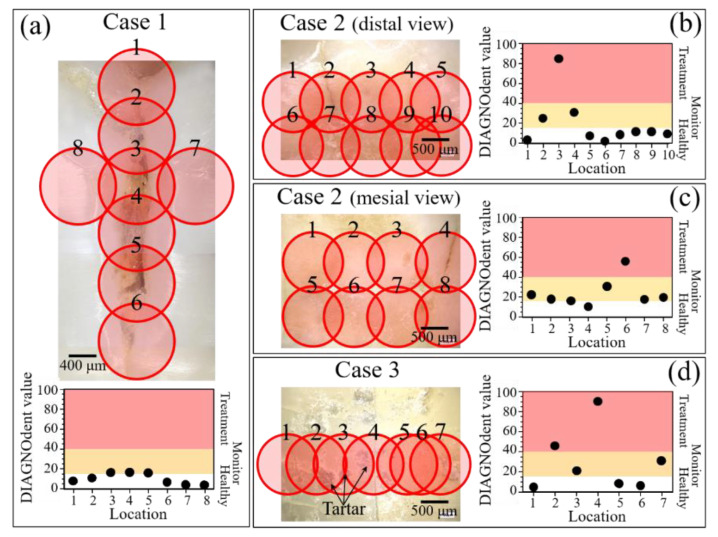
LF analyses of (**a**) Case 1, (**b**) Case 2 (distal view), (**c**) Case 2 (mesial view), and (**d**) Case 3; line scans were performed on each tooth surface and the measurement points were defined as numbers. Graphs in each section represent the severity of the caries according to the DIAGNOdent value expressed as a number between 0 and 100 [20,21,22,23,24,25].

In this section we assess the performance of a commercially available LF pen device. In Case 1, no caries requiring treatment was detected. In Case 2, LF assessments identified Location 3 (distal view) and Location 6 (mesial view) as requiring invasive treatment, with some neighboring locations requiring monitoring for future caries development) (Figure 7b,c). The biased LF assessment in Case 3 was clearly due to the presence of tartar on the tooth surface (cf. arrows in Figure 7d). Although the maker of the LF device recommends preliminary removal of tartar prior to caries assessment, the bias potentially induced by the presence of tartar residues at the measurement location clearly adds a component of uncertainty to caries diagnosis by LF, as the technique still requires a dentist’s subjective assessment for confirmation and thus lacks objectivity.

### 3.3. Raman Spectroscopic Analyses

**Figure 8:**
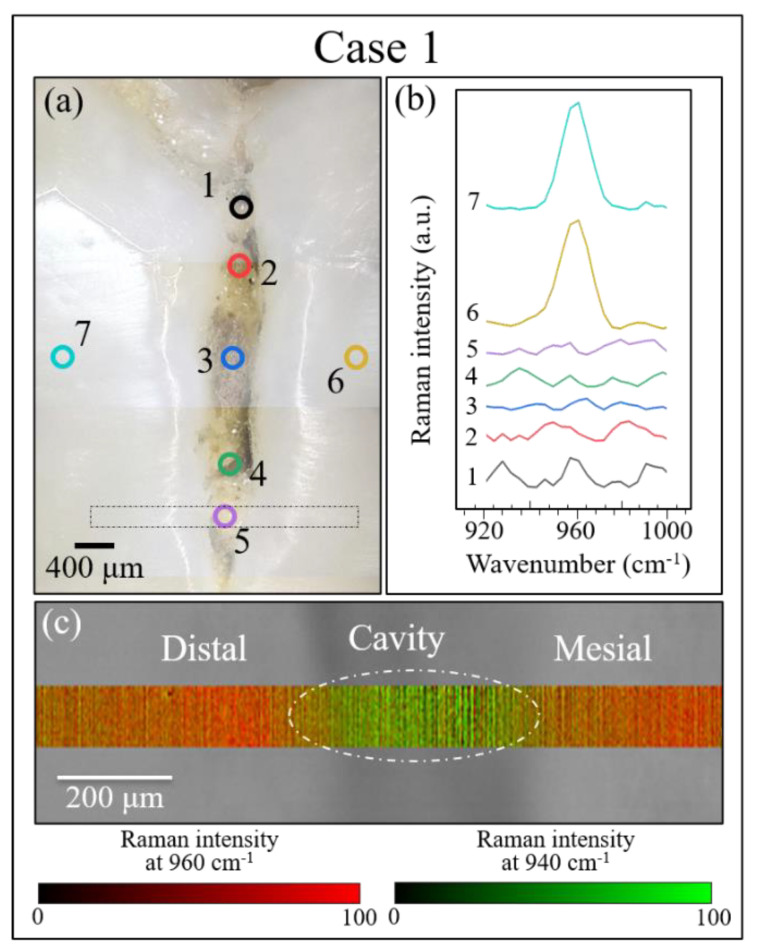
Raman analysis of the tooth sample in Case 1: (**a**) optical image (100× lens) showing locations (1~7) selected for point analysis, (**b**) recorded Raman spectra (10× lens) at the selected locations (cf. labels) in the spectral region around 960 cm^−1^, and (**c**) Raman map corresponding to the area indicated by the rectangular inset in (**a**).

**Figure 9:**
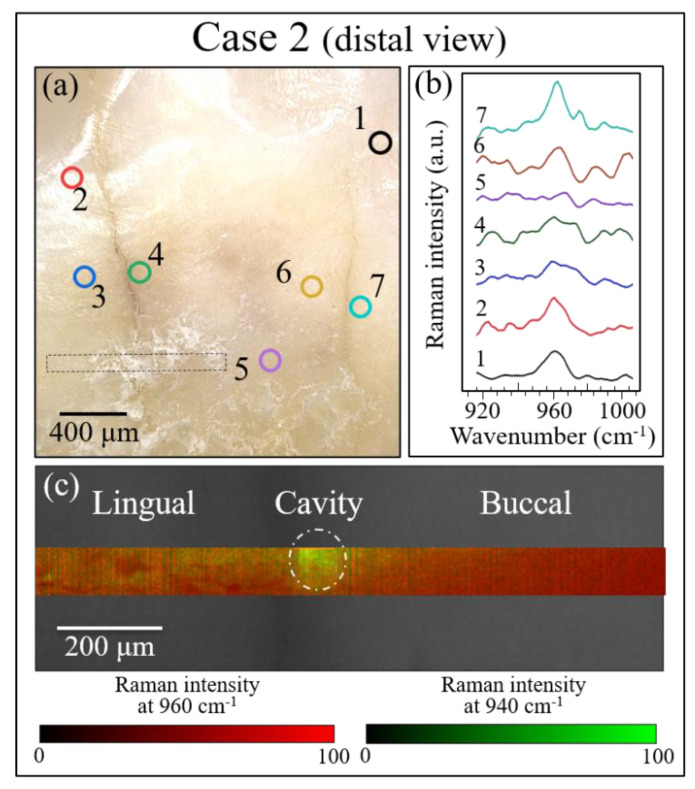
Raman analysis of the tooth sample in Case 2: (**a**) optical image (100× lens) showing locations (1~7) selected for point analysis, (**b**) recorded Raman spectra (10× lens) at the selected locations (cf. labels) in the spectral region around 960 cm^−1^, and (**c**) Raman map corresponding to the area indicated by the rectangular inset in (**a**).

**Figure 10:**
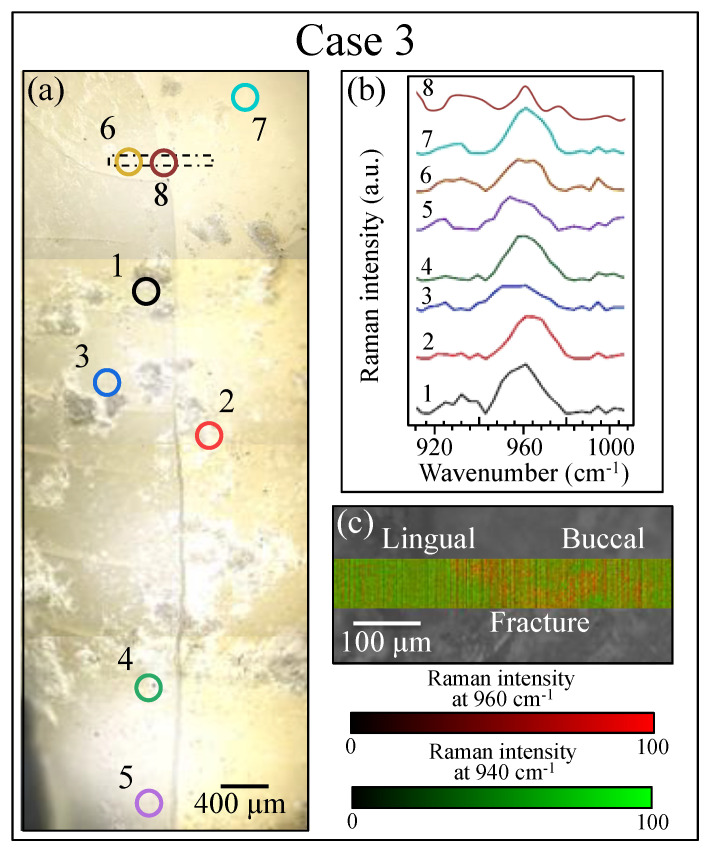
Raman analysis of the tooth sample in Case 3: (**a**) optical image (100× lens) showing locations (1~8) selected for point analysis, (**b**) recorded Raman spectra (10× lens) at the selected locations in the spectral region around 960 cm^−1^, and (**c**) Raman map corresponding to the area indicated by the rectangular inset in (**a**).

Figure 8, Figure 9 and Figure 10 show the results of our Raman analyses on Cases 1, 2, and 3, respectively. This is a summary of the results for the same samples and locations as the LF measurements shown in Figure 7. As a preliminary procedure on all samples, several point locations were separately analyzed, some around the fracture/cavity areas and others, as reference points, far away from them. The locations of point analysis are shown on optical images in section (a) of each of these three figures, while section (b) of each figure (cf. labels) shows the recorded spectra for the spectral region around the 960 cm^−1^ Raman band (i.e., the band representing the symmetric stretching of the PO_4_^3^^−^ tetrahedra in hydroxyapatite [26]). Through monitoring individual spots, the following preliminary judgments could be made by examining the intensity and the morphology of the 960 cm^−1^ Raman signal:

Case 1: Unlike areas far away from the crack (cf. locations 6 and 7 in Figure 8a), which presented a relatively strong and sharp signal at 960 cm^−1^, locations 1–5 along the crack showed a significantly lower signal (or no signal) for the hydroxyapatite band (cf. Figure 8b).

Case 2 (distal view): In this sample, the spot at location 5 showed almost no Raman signal at 960 cm^−1^, which clearly indicated an alteration of the hydroxyapatite tooth structure (as quantitatively discussed in Section 4). Conversely, the strongest signals were detected at points 1, 2, and 7, which were control spectra taken at locations slightly more distant from the visible cracks (cf. Figure 9a,b).

Case 3: In this sample, alterations of the Raman signal from the tooth mineral component were far less pronounced than those observed in Cases 1 and 2; no locations with a complete absence of the Raman signal could be observed at any of the monitored locations (cf. Figure 10a,b). In the neighborhood of the fracture path, only a slight decrease in intensity or shift of the 960 cm^−1^ Raman band could be observed. The monitored locations in this sample were all in the dentin area, which contains a lower fraction of hydroxyapatite as compared to enamel.

**Figure 11:**
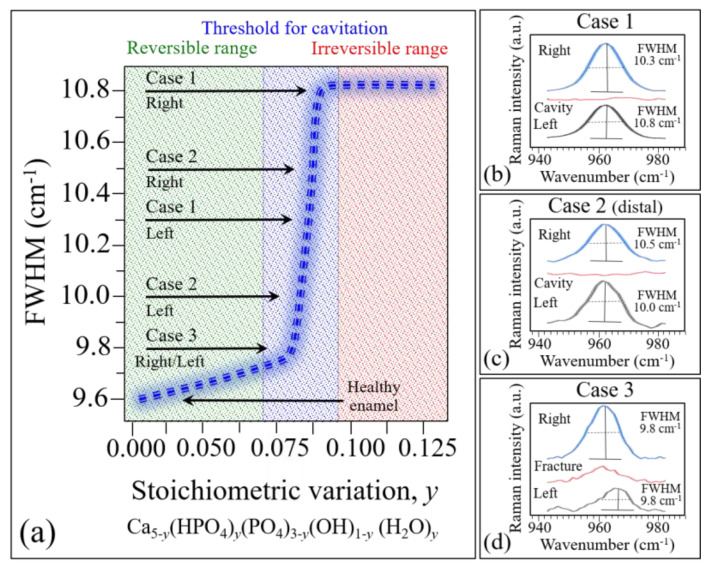
(**a**) Relationship between the FWHM of the 960 cm^−1^ Raman band of hydroxyapatite and the stoichiometry parameter y from Ref. [7] (cf. Figure 11b and related text in that reference); and the average Raman spectra from three different areas (cf. labels) of the tooth samples in Cases 1 (**b**), 2 (**c**), and 3 (**d**); the FWHM of average bands are analyzed in (**a**). The terms “right” and “left” in (**b**) refer to the distal and mesial sides of the cavity, respectively, inside the cavitated area encircled in inset to Figure 8c. Similarly, the same terms in (**c**) and (**d**) refer to buccal and lingual sides of the cavity, respectively, with reference to the cavitated areas encircled in Figure 9c and Figure 10c, respectively.

Notably, for Case 1, the LF method did not detect any location that required invasive treatment (Figure 7a), while the Raman assessments indicated an advanced state of disease in the sub-surface of the discolored zone (cf. Figure 8c) and a state of disease at the threshold of irreversibility on both the mesial and distal sides of the buried cavity (as we shall see later). In Case 2, Raman assessments in the same zone fully agreed with the LF diagnosis with respect to both the cavity zone (cf. Figure 9c) and its neighboring zones at the threshold of irreversible cavitation (cf. Figure 11). In Case 3, conversely, the LF scan across the cracked zone in the dentin detected two locations in need of invasive treatment (Figure 7d), while the Raman scan detected no caries and a dentin structure that remained within the stage of reversible demineralization/remineralization (cf. Figure 9c) and as we shall see later).

The rectangular areas outlined in dotted lines in section (a) of each figure (Figure 8, Figure 9 and Figure 10) were mapped with the Raman probe with a single-micron spatial resolution over a total area of ~10^3^ μm^2^. The results are shown in sections (c) of each figure (Figure 8, Figure 9 and Figure 10). Raman mapping clearly confirmed the possibility of recognizing diseased areas by their abnormal intensity of the Raman band of hydroxyapatite, as this signal systematically tended to disappear in zones with altered hydroxyapatite structure such as cavities. In Case 1 (Figure 8c), the band localized at 960 cm^−1^ was conspicuously absent in the area corresponding to the cavity (cf. circled area) indicating a lack of mineral phase (mapped in red color; cf. scale under the map), while only the signal from collagen at ~940 cm^−1^ could be detected in this area (mapped in green color). A similar result was obtained for the sample in Case 2 (distal view) (cf. Figure 9c), in which the circled cavity area was depleted of the apatite Raman signal (red color) and presented only organic signals (green color). In the Case 3 sample (Figure 10c), the Raman map across the crack path in the dentin zone was inherently different from the Raman maps obtained for the samples in Cases 1 and 2. The predominant signal was that of collagen (green color), due to the difference in structure of dentin as compared to enamel. Yet the 960 cm^−1^ apatite signal was clearly detected, and there was no location in which the apatite signal completely disappeared. Accordingly, the Raman map suggested no or conspicuously less severe cavitation both at the surface and in the sub-surface of the Case 3 sample. The most important outcome of our Raman mapping analyses was that they allowed us to visualize diseased zones (i.e., zones in a high state of demineralization) that were not detectable by visual inspection because they were buried in the sub-surface. Most importantly, these diseased zones systematically coincided with the cavitated zones observed by micro-CT scanning experiments.

## 4. Discussion

### 4.1. Linking the Raman Spectrum to the Severity of the Cavitated Zone

In previous works [1,6,7], we have systematically and quantitatively monitored the evolution of tooth enamel demineralization according to the chemical formula, Ca_5-y_(HPO_4_)_y_(PO_4_)_3−y_(OH)_1−y_ (H_2_O)_y_. This is a generally valid chemical representation of the enamel apatite structure in the presence of a fraction *y* of Ca vacancies. This chemical equation is independent of the way Ca-vacancies form and of the local environment. We demonstrated that this equation maintains its validity in the early stages of tooth demineralization when, conceivably, only screw-axis Ca ions are removed. The structural disorder due to the formation of Ca vacancies (and represented by the stoichiometry parameter, *y*) is quantitatively related to the broadening of the 960 cm^−1^ apatite band, as both demineralization experiments and phonon density of states calculations have shown [7]. Note that the vibrational band at 960 cm^−1^ is not directly related to chemical bonds involving calcium; rather, the formation of Ca-vacancies creates a state of local disorder in the crystalline structure of hydroxyapatite, which alters the vibrational frequency of PO_4_^3^^−^ tetrahedra. Such fluctuations in vibrational energy in turn lead to the observed band broadening. The relationship between the width of the 960 cm^−1^ Raman band and the stoichiometry parameter y is shown in Figure 11a. According to this graph, the full width at half maximum (FWHM) increases linearly with increasing Ca^2+^ off-stoichiometry in the hydroxyapatite structure up to a value of 0.08 (corresponding to the presence of ~0.75 mol.% of Ca^2+^ vacancies). In this initial interval, the stoichiometry fluctuations are still reversible and can be healed through remineralization. The value *y* = 0.08, however, represents a threshold value above which an abrupt increase in FWHM up to ~10.8 cm^-1^ occurs until the parameter *y* reaches approximately the value 0.1. Above this threshold, the stoichiometric damage is irreversible: a cavity can form and increase in volume over time. Note that the FWHM of the 960 cm^−1^ Raman band in healthy enamel and that in a single perfectly stoichiometric hydroxyapatite crystal have been measured at ~9.8 and 9.6, respectively [1]. The FWHM of the 960 cm^−1^ band does not broaden further at *y* > 0.1, but its band intensity abruptly decreases and, above a value of *y* = 0.125, the Raman signal completely disappears. We shall henceforth use the dependence in Figure 11a to interpret the present Raman data.

Average spectra were computed from experimental maps collected in three different areas of each of the three studied samples, as shown in Figure 11b–d. According to the graph in Figure. 11a, the FWHM of average bands can be used to identify the stoichiometric state of a tooth and, accordingly, the severity of the disease in terms of its reversibility (i.e., the possibility of remineralization). According to this criterion, the average stoichiometric state in the right area (on the mesial side) of Case 1 was the most severe, as the tooth decay had reached an irreversible stage (cf. inset in Figure 11a), while a similar evaluation of the left area (on the distal side) revealed a less severe disease state, as the tooth decay was yet at the threshold of irreversibility (cf. Figure 8c) and Figure 11a,b). Note that the evaluated areas were adjacent to the cavity, while the cavity area itself showed no apatite Raman signal, as expected due to the high level of damage (cf. average spectrum in Figure 11b). The Case 2 sample exhibited a similar pattern, with no signal in the cavity zone of the disease, and clear off-stoichiometric states at the threshold for irreversibility in the adjacent right (buccal side) and left (lingual side) zones (cf. Figure 9c and Figure 11a,c). Case 3 was the least severe among the three cases, as shown by its average spectra depicted in Figure 11d. The Raman signal was still present in the cavity zone, though it was very weak, and its broadness indicated that the tooth decay had progressed into an irreversible state. In the adjacent right (buccal side) and left (lingual side) zones, however, the stoichiometric damage was yet at the limit of reversibility (cf. Figure 10c and Figure 11a,d). An interesting detail was the comparison between Location 8 of Case 3 in Figure 10 and the correspondent Location 4 in Figure 7d. The high fluorescence signal recorded in the latter would suggest a highly cavitated condition in need of treatment. However, the Raman spectrum recorded at this position (as shown in Figure 10b) was among the sharpest (and thus the structurally healthiest) ones recorded in this study. We interpreted this discrepancy as a bias of the fluorescence measurement, which was influenced by the presence of tartar residues in the measurement zone and in its neighborhood.

Mapping of the hydroxyapatite structure at the molecular scale was performed in both enamel and dentin and was shown to be useful for locating buried cavities in zones where visual evaluation is difficult. Leaching of Ca^2+^ ions in decayed areas and the consequent formation of cavities are clearly linked to the altered vibrational behavior of PO_4_^3-^ tetrahedra in the hydroxyapatite structure, whose strongest signal corresponded to the P-O bond stretching at ~960 cm^−1^. Compared to that in healthy enamel, the P-O stretching Raman band clearly decreased in intensity and increased in breadth with increasing severity of tooth decay. A previously developed algorithm allowed us to quantitatively quote an off-stoichiometric chemical equation linking the FWHM of the 960 cm^−1^ Raman band to the chemistry of enamel demineralization and ultimately to the severity of sub-surface cavity development. Note that, although Raman data for healthy teeth samples generally display a bandwidth at ~9.6 cm^−1^ or less, this value might show a dependence on age, gender, and race. It, therefore, needs to be calibrated on individual patients at location of healthy enamel or dentin, or possibly be related to data from the same patient in time lapse.

### 4.2. The Usefulness of the Raman Approach in Preventive Dentistry

Enamel and dentine structures can be evaluated promptly by Raman spectroscopy at any point in their gradual evolution from a healthy state through increasing levels of demineralization related to the effect of incorrect alimentary habits or other pathological circumstances. The risk of tooth cavity formation could then be quantitatively classified by analyzing, at the molecular scale, the vibrational response of the main apatite scaffold present in the structures of both tooth enamel and dentine. The spectroscopic findings presented in this paper clearly demonstrate that the Raman method is useful not only as a diagnostic tool but also as a preventive one.

Progress in diagnostic dentistry requires more accurate risk assessment, earlier detection, and a better understanding of the biological development of dental caries. Recent efforts have focused on the prevention of tooth decay rather than surgical removal [27]. New diagnostic strategies often fail to work as well as expected in the short term because clinicians lack experience in using these new tools as well as in interpreting their findings; new tools also present cost and safety issues. It should be noted that the micro-radiographic [28], fiber-optic transillumination [29], laser fluorescence [30], and electrical conductance methods [31] are not yet considered improvements on the traditional methods, because their results are not generalizable, because their ultimate outputs are prone to scatter, or because they are insufficiently safe [32,33]. Raman spectroscopy could represent a breakthrough as it outperforms all of these other methods in terms not only of performance but also of safety and cost, and could thus be the key to improving the state of oral health in modern societies, which is increasingly depressed due to newly developed eating patterns.

### 4.3. The Limitations of This Study

We offered here some clear proofs that the assessment of early enamel demineralization and dental caries is possible by Raman imaging. However, a dedicated instrument for intraoral assessments in medical practice is not yet available in the market. Such handheld instrument should be capable to provide both high sensitivity and sufficient spectral resolution for converting the Raman signal into a reliable and factual medical assessment. This is yet work in progress. Moreover, regarding the type of tooth, we have given extensive descriptions in previous published papers on the same topic (cf. Refs. 1, 6, and 7). However, assessments of deciduous teeth have yet to be performed. Although Ref. 7 suggests that it could be possible to design an experimental procedure addressing the stoichiometry parameter, *y*. This item has yet been beyond the achievements of the current study. Finally, the present study, as well as the previously published ones (as mentioned above), only refers to teeth collected on Japanese individuals. Note also that, according to ethical constrains in Japan as related to the research ethics committee, which also includes privacy issues, it is not allowed to link the obtained teeth to the age and/or the gender of the donor.

## 5. Conclusions

We believe that Raman methodology and related diagnostic algorithms, as applied in this study, could represent a breakthrough in dentistry. Raman spectroscopy could not only open the way to rapid and safe in-vivo assessments of enamel and dentin quality but also provide a unique path to developing reliable procedures for caries risk prediction.
